# Role of Laccase and Low Molecular Weight Metabolites from *Trametes versicolor* in Dye Decolorization

**DOI:** 10.1100/2012/398725

**Published:** 2012-04-01

**Authors:** Diego Moldes, María Fernández-Fernández, M. Ángeles Sanromán

**Affiliations:** Department of Chemical Engineering, University of Vigo, Isaac Newton Building, Lagoas-Marcosende s/n, 36310 Vigo, Spain

## Abstract

The studies regarding decolorization of dyes by laccase may not only inform about the possible application of this enzyme for environmental purposes, but also may provide important information about its reaction mechanism and the influence of several factors that could be involved. In this paper, decolorization of crystal violet and phenol red was carried out with different fractions of extracellular liquids from *Trametes versicolor* cultures, in order to describe the role of laccase in this reaction. Moreover, the possible role of the low molecular weight metabolites (LMWMs) also produced by the fungus was evaluated. The results confirm the existence of a nonenzymatic decolorization factor, since the nonprotein fraction of the extracellular liquids from cultures of *T. versicolor* has shown decolorization capability. Several experiments were performed in order to identify the main compounds related to this ability, which are probably low molecular weight peroxide compounds.

## 1. Introduction

Laccases (EC 1.10.3.2) are multicopper oxidases mainly involved in phenolics oxidation. Actually they can not only catalyze mono-, di-, and polyphenols, but also aminophenols and methoxyphenols, even aromatic amines and ascorbate. Moreover, the combination of laccases with several low molecular weight substrates (mediators) may lead to an increase of the oxidation power of the enzyme and, in addition, the range of substrates that could be oxidized [1, 2].

Laccases are mainly produced by wood rooting fungi, although several bacteria, insects, and plants can also produce them. The most known and interesting fungal laccases are the extracellular ones due to the high production achieved and the easy recovery. The versatility and activity of extracellular fungal laccases have enabled to undertake many studies regarding their possible industrial applications such as potential bioremediation agents for organic pollutants, biosensors, biobleaching of paper pulp, organic synthesis and many others [3, 4]. Among them, one of the most explored subjects is their capability for dye decolorization [5–7]. Dyes, chemical compounds mainly used in textile industry, are usually organic compounds belonging to several families of chemicals depending on their chemical structure (azo, phthalocyanine, sulphur, nitro, antraquinone, etc.) or the way of application (acid, mordant, reactive, direct, disperse, etc.). The diverse composition of dyes usually produces recalcitrant textile effluents with two key characteristics: toxicity and color. The toxicity of textile effluents depends on the chemical structure of dyes and is usually high at low dye concentrations, while color of textile wastewater is due to the chromophoric groups of dyes. This color modifies the physical properties of the water bodies and may cause alterations on their ecological equilibrium.

In the last years, dye decolorization by laccases has been proposed as a possible treatment of textile wastewater due to their oxidation power and relative unspecificity, which allows to oxidize different chemical structures [8]. Moreover, the decolorization studies have also been proposed as model studies to investigate the potential degrading capability of this enzyme or even of several fungal strains [9–11]. As it was mentioned above, dyes have similar chemical structures than other pollutants, but their degradation can be easily monitored by means of coloration decrease of the analyzed system. This idea was used for several authors for searching fungal strains with potential ability of pollutants degradation, by means of analyzing the decolorization of model dyes in petri dish cultures, where the decolorization is evident when it takes place [12]. On the other hand, decolorization with laccase *in vitro*, in absence of biomass, was also performed as an indicator of the degrading potential of this enzyme, even some correlation experiments between decolorization capability and degradation of polycyclic aromatic hydrocarbons could be found in the bibliography [9].

In this paper, laccase produced by *Trametes versicolor* was selected in order to test the decolorization of two chemically different dyes. The decolorization study was carried out in presence and absence of the low molecular weight metabolites (LMWMs) produced by the fungus, in order to determine their role in the enzymatic dye decolorization. In addition, taking into account that lignocellulosic waste sources are well-known inductors of laccase production and may also modify the composition of the extracellular liquid produced by the fungus, where laccase and the LMWM are present, several experiments with different fractions of extracellular liquids from *T. versicolor *cultures in presence of different lignocellulosic residues were performed and the role of enzymatic and nonenzymatic factors in the decolorization of the selected dyes is discussed.

## 2. Material and Methods

### 2.1. Laccase Production in Liquid Medium

The fungus *Trametes versicolor* CBS 100.29 (CECT 20148) was maintained in petri dishes cultures at 4°C with a basic culture medium composed of (per l): 20 g glucose, 15 g agar-agar, 2 g malt extract, 5 g peptone, 1 g KH_2_PO_4_, and 0.5 g MgSO_4_·5H_2_O. One plug of 5 mm diameter from this maintenance cultures was used to grow the fungus in fresh petri dishes with a similar culture medium (10 g/L glucose, 15 g/L agar-agar, and 3.5 g/L malt extract). After 10 days of growing at 37°C, 3 plugs of 5 mm diameter were removed from the petri dishes and used to inoculate the liquid medium based on that described by Tien and Kirk [13] and consisting of (per l): 10 g glucose, 0.2 g ammonium tartrate, 0.2 L of sodium acetate/acetic acid buffer 0.1 M pH 4.5, 5 mL Tween 80, 142 mg nitrilotriacetate, 1.46 g MgSO_4_·7H_2_O, 40 mg MnSO_4_·3H_2_O, 70 mg NaCl, 7 mg FeSO_4_·7H_2_O, 13 mg CoCl_2_·6H_2_O, 132 mg CaCl_2_·2H_2_O, 7 mg ZnSO_4_·7H_2_O, 11 mg CuSO_4_·5H_2_O, 1 mg AlK(SO_4_)_2_·12H_2_O, 1 mg H_3_BO_4_, Na_2_MoO_4_·2H_2_O, 2 g KH_2_PO_4_, and 0.1 mg thiamine-HCl. Fernbach flasks of 1.8 L were used to carry out the cultures with 100 mL of the described liquid medium. The cultures were incubated at 30°C in static condition for 10 days. In addition 0.3 mM 2,5-xylidine was added as laccase production inducer.

After culture, the liquid was consecutively filtered by 0.45 and 0.2 *μ*m to remove biomass. Then several fractions of this extracellular liquid were obtained as explained later in this section.

### 2.2. Laccase Production with Lignocellulosic Residues

Several lignocellulosic residues were employed as inducers for laccase production in *T. versicolor* cultures:

- wheat straw, provided by San Martin S.A. (Ourense, Spain), was cut for obtaining pieces of 7 mm as average;
- corn cobs, provided by local producers, were cut, sieved, and the particles of 7 mm diameter were selected;
- grape stalks and grape seeds, provided by Bodega Vilariño (Cambados, Spain). The grape stalks were cut to 7 mm pieces, while the grape seeds did not suffer any physical modification.

All the lignocellulosic residues were washed with tap and distilled water, then dried at 60°C and sterilized in an autoclave (121°C for 20 minutes) before use.

The cultures were performed in 250 mL erlenmeyer flasks with enough solid substrate to cover the bottom surface of the flasks. 100 mL of liquid medium, prepared as previously commented, were added and the incubation was performed in a shaker in dark conditions, at 30°C and 150 rpm.

After 25 days of culture, the liquid medium was recovered and three enzyme fractions were used on decolorization experiments: extracellular liquid (EL), enzymatic crude (EC), and ultrafiltration residue (UR). They were obtained as follows:

EL: it is the culture medium after consecutive filtration by 0.45 and 0.2 *μ*m;
EC: aliquots of EL were ultrafiltered with a membrane of 10 KDa molecular cutoff. The protein concentrate obtained was then dialyzed with a 10 KDa membrane in acetate buffer (10 mM pH 5);
UR: ultrafiltration residue generated in the ultrafiltration process, which contained the nonprotein fraction of EL and consisting of LMWM with molecular mass lower than 10 KDa.

**Figure 1:**
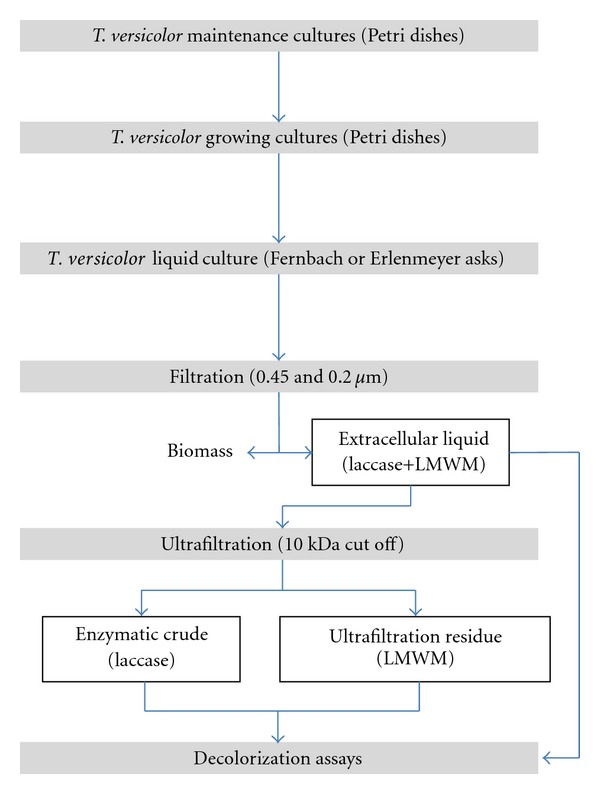
Experimental flow chart of the production of the enzymatic fractions for accomplishment of decolorization assays: extracellular liquid (EL), enzymatic crude (EC), and ultrafiltration residue (UR).

A summary of the production of the enzyme fractions is presented in Figure 1.

### 2.3. Laccase Activity Analysis

Laccase activity was determined spectrophotometrically by following the oxidation of 2,2′-azinobis(3-ethylbenzothiazoline-6-sulfonate) (ABTS) at 436 nm (*ε* = 29300 M^−1 ^cm^−1^). Assay mixture of 1.5 mL contained: 25 mM succinic acid, 5 mM ABTS, and 200 *μ*L of sample. One unit of laccase activity was defined as the amount of enzyme required to oxidize 1 *μ*mol of ABTS per minute at the analysis conditions: 23°C and pH 3.

### 2.4. Decolorization Studies

Two structurally different dyes were studied: phenol red (PR) and cristal violet (CV). Their decolorization was carried out in a mixture of 2.5 mL consisting of 75 *μ*M PR or 15 *μ*M CV, 10 mM acetate buffer (pH 4.5) and the amount of enzyme fraction required to attain 1 U/mL of final laccase activity in the reaction mixture. When UR was used as the enzyme fraction, same volume of the original EL was employed. The decolorization was carried out at 30°C in dark condition and analyzed by monitoring the absorbance spectrum of both dyes (350–600 nm for PR and 400–650 for CV) with a Spectronic Unicam spectrophotometer (model Helios Beta) for a maximum of 6 days. Assays were undertaken in triplicate with a resulting standard deviation lower than 5%. Control experiments without enzyme fraction were conducted in parallel.

### 2.5. Organic Acids Analysis

The organic acids content in each EL of the cultures employed was analyzed by a Jasco HPLC equipped with a UV detector and a Rezex column 10u 8% H (300 × 7.8 mm), with isocratic elution and operation at room temperature. The mobile phase consisted of 0.01 M H_2_SO_4_ with flow rate as 0.5 mL/min. 5 *μ*L of each sample were injected in the HPLC and detection was performed at 210 nm. The organic acids analyzed by this method were citric, oxalic, malonic, and acetic acid. Peak identity was accomplished by retention time comparison of the pure organic acids.

## 3. Results and Discussion

### 3.1. Decolorization by Different Laccase Fractions: Enzymatic and LMWM Roles

The production of several metabolites with oxidation power has been detected in previous works [14, 15]. For this reason, the main aim of this study is to determine the role of LMWM produced as possible oxidants in the decolorization processes.

Three fractions (EL, EC, and UR) from *T. versicolor* cultures carried out in liquid medium, as previously explained, were tested in order to analyze the role of the enzyme and LMWM in the decolorization of two structurally different dyes.

**Figure 2:**
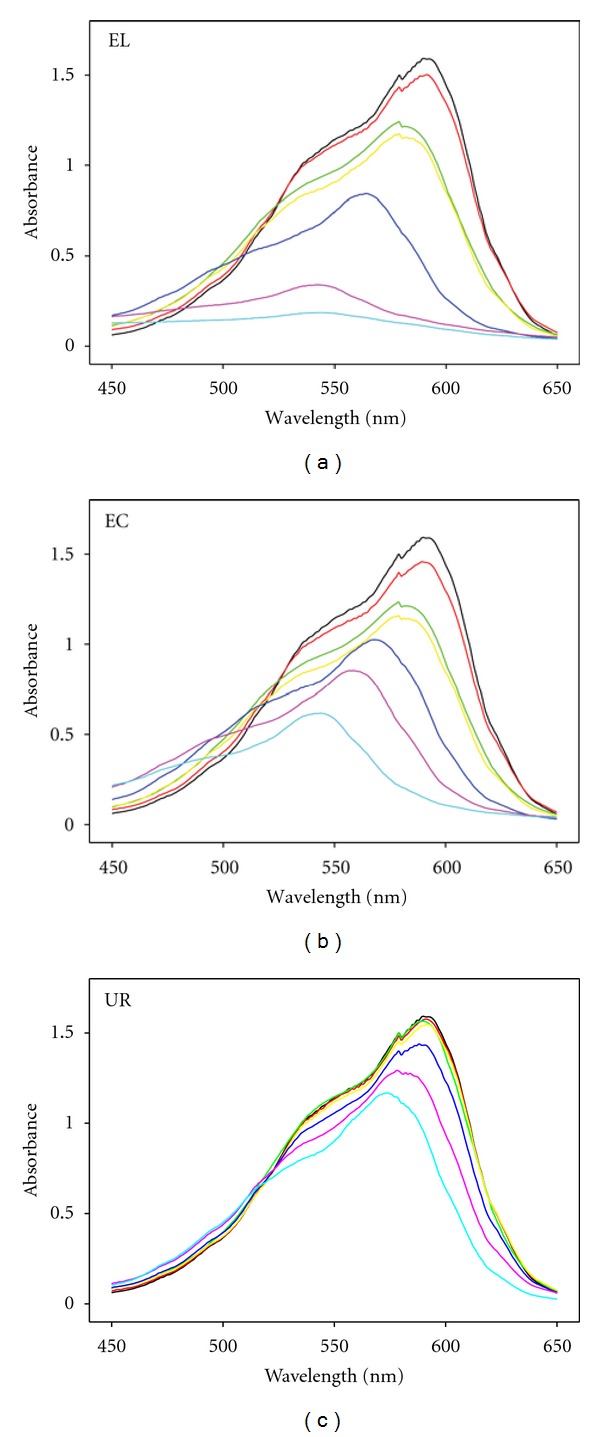
Decolorization of CV by three enzyme fractions (EL: extracellular liquid; EC: enzymatic crude; UR: ultrafiltration residue). Time of reaction: 0 h (black), 0.5 h (red), 2 h (light green), 4.5 h (yellow), 1 d (blue), 3 d (violet), 6 d (cyan).

In Figure 2, the evolution of the visible absorption spectrum of the CV dye, by the three laccase fractions, is showed. It is noticeable that in the control experiment without enzyme fraction did not achieve any decolorization (data not shown). However, EL and EC showed similar decolorization capability, although EL was more effective after one day of treatment. On the other hand, due to UR fraction is the nonenzymatic fraction, the expected behavior was similar to the profile shown in control experiments, however surprisingly decolorization was detected in these experiments, which contains the LMWM lower than 10 KDa. This result shows that laccase is not the only oxidation agent from the extracellular liquid of cultures of *T. versicolor*, since LMWM in absence of enzymes can perform the decolorization of CV. Therefore, decolorization score performed by the EL could be explained as an additive effect of the decolorization achieved by laccase and the LMWM.

**Figure 3:**
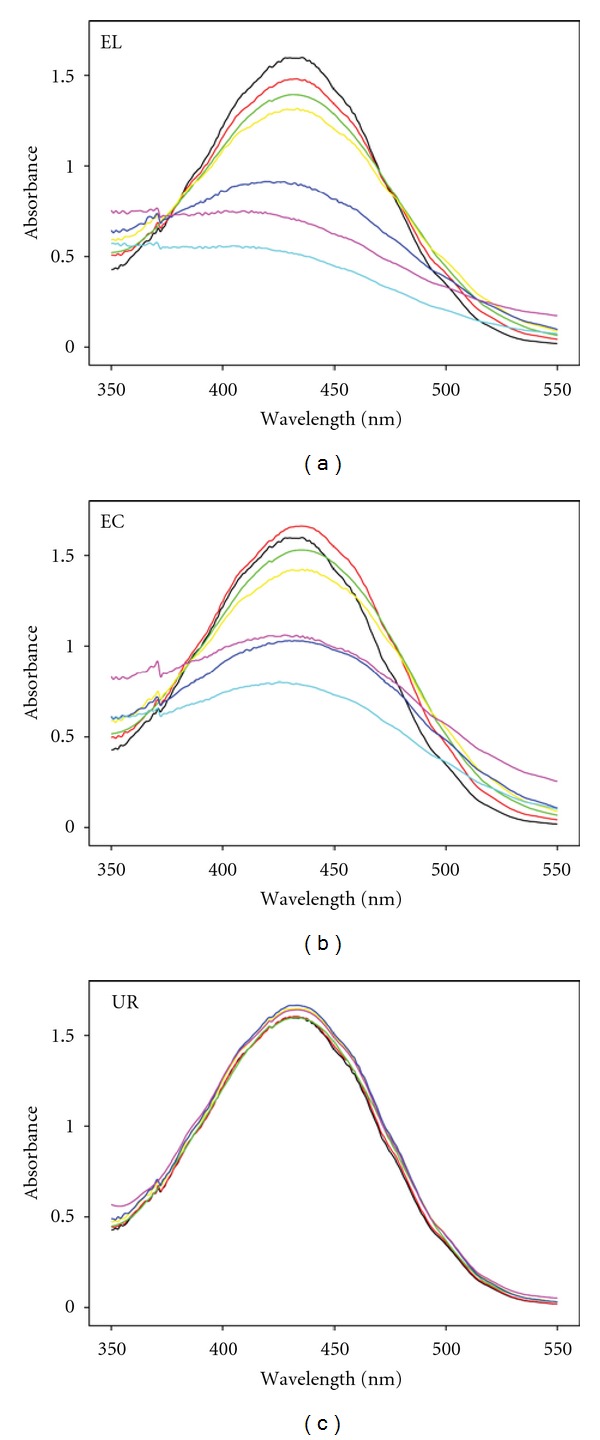
Decolorization of PR by three enzyme fractions (EL: extracellular liquid; EC: enzymatic crude; UR: ultrafiltration residue). Time of reaction: 0 h (black), 0.5 h (red), 2 h (light green), 4.5 h (yellow), 1 d (blue), 3 d (violet), 6 d (cyan).

Same experiments accomplished with PR as dye, instead of CV, showed some differences (Figure 3), since when UR was added any detectable modification of the dye was detected. Therefore, the presence of laccase is an essential factor for PR decolorization. However, EL presented a better performance in PR decolorization than EC, suggesting that the LMWM may be indirectly involved in enzymatic PR decolorization, possibly attaining the stabilization of the enzyme. This stabilization effect of the LMWM has been previously described [16]. However, analyzing the decolorization rate for the first 2 hours of reaction, EL and EC experiments showed a very different decolorization rate, which suggest another role of the LMWM.

Based on these results, two possible roles, besides the enzyme stabilization, could be assigned to LMWM: working as mediators or dye oxidation capability. The former possibility has been described for several authors [17, 18], while the latter has been mentioned above in the case of CV decolorization by the UR fraction. The capability of dye oxidation by the UR may be attained by two main mechanisms: hydroxyl radicals and peroxides, since both may be produced by wood rooting fungi. The hydroxyl radicals have a short life-time to remain active after the process of UR fraction; therefore the main factor in the dye decolorization capability seems to be the presence of peroxides. Peroxides may oxidize some chemical structures by themselves but they could also promote oxidation by means of hydroxyl radicals formation by the mechanism of Fenton reaction in presence of Fe^2+^. Several authors have proposed the Fenton-like reactions as an important nonenzymatic mechanism for degradation of lignin, pollutants and dye decolorization by ligninolytic fungi [19–21]. In order to verify this hypothesis new decolorization tests were designed in presence of catalase.

Catalase has the ability to react with peroxides to produce water. Therefore, after a semiquantitative peroxide determination with peroxide strips, catalase was added to the decolorization test tubes in the necessary concentration to eliminate the peroxides from the UR and EL. The results from these experiments (Table 1) showed not only the absence of CV decolorization by the action of UR, but also a higher rate of decolorization of EL in comparison with no catalase addition. Similar results were obtained in the study of PR dye, in which high decolorization values were detected in experiments carried out with EL and catalase, although the initial rate of decolorization was reduced. 

Therefore, the presence of peroxides seems to be responsible of the dye removal capability of UR, but additionally the LMWM with inactivated peroxides can contribute to the dye degradation, by means of their possible role as mediator or by enzyme stabilization, as we previously hypothesized. An increase of initial decolorization rate would suggest the presence of a mediator compound in the LMWM fraction. However, considering that this effect was not clearly produced after catalase addition and the decolorization level attained after 1 day or more time was increased in comparison with experiment without catalase, the enzymatic stabilization hypothesis appears to be the most possible one. This result is in agreement with the stabilization effect of catalase observed with other ligninolytic enzymes [22]. Two completely different decolorization processes seems to coexist: decolorization by peroxides with possible generation of radicals, and enzymatic decolorization. The latter can be affected by the peroxides as these chemicals may inactivate the enzyme. 

### 3.2. Decolorization with Several Laccase Sources: Potential Decolorization by LMWM

**Figure 4:**
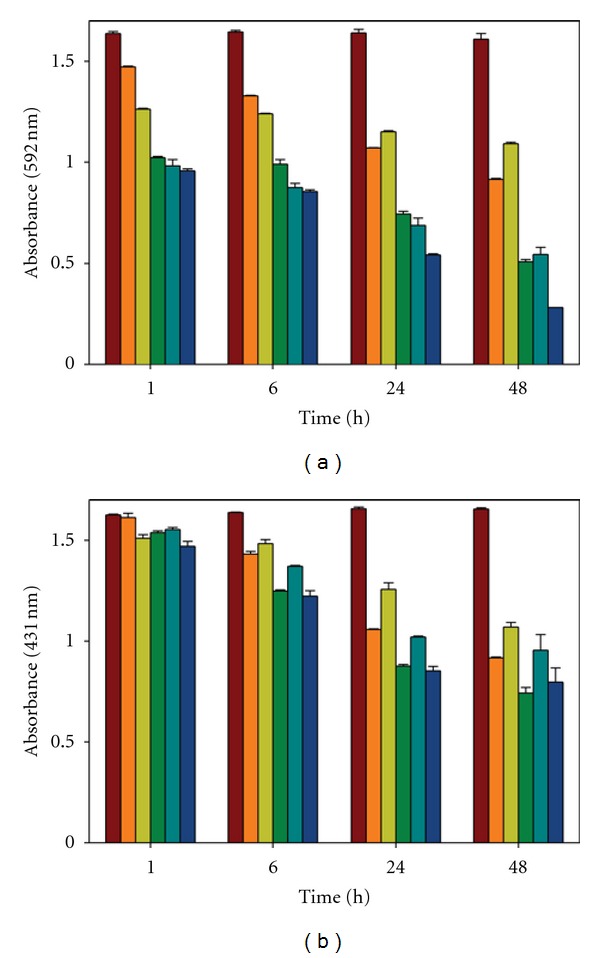
Decolorization of CV (a) and PR (b) by extracellular liquid from cultures of *T. versicolor* performed with several lignocellulosic residues: (maroon) control (noninoculated liquid medium), (orange) culture with no additive, (light green) corn cob, (dark green) wheat straw, (teal) grape seeds, and (navy blue) grape stalks. Decolorization of CV and PR was followed by decrease in absorbance at 592 and 431 nm, respectively.

Lignocellulosic materials comprise a broad range of wastes from agricultural, food, and forest industry that can stimulate laccase production [23–25]. Therefore, the interest of addition of these materials is diverse: induction of laccase production, use of agriculture residues (waste reduction and valorization), possible reduction of chemicals in liquid medium and production of extracellular liquid with different composition. This latter effect has a great interest in decolorization studies, since the modification of the enzymatic and the nonenzymatic composition (LMWM) of the extracellular liquids produced, may lead to different decolorization capabilities. In this study, the decolorization ability of several enzymatic fractions was studied after production of laccase by culturing *T. versicolor* in presence of different lignocellulosic residues: corn cobs, wheat straw, grape stalks and grape seeds. In Figure 4, the decolorization profiles of CV and PR by action of EL from the cultures previously described are showed. In these experiments, different ELs with the same laccase activity were used. The results obtained in these decolorization tests show the expected different decolorization capability for each EL fraction. In the decolorization experiments of CV, most of them improved the result obtained with the EL from the culture without additive. However, in the experiments performed with PR dye, the differences were not remarkable, indicating that the different composition of ELs has not a key importance and that the enzyme is the main factor in the decolorization reaction.

The decolorization capability of the several ELs could be ordered as follows:

- CV decolorization: grape stalks > wheat straw = grape seeds > no additive > corn cobs;
- PR decolorization: grape stalks = wheat straw > grape seeds > no additive > corn cobs.

To explain the previously mentioned differences, the decolorization capability of the protein and non-protein fractions of each EL, that is, EC and UR, respectively, was tested with CV dye. In these new experiments the corn cob EL was discarded, due to the lack of positive results, and the time of treatment was reduced to just one hour, considering that the first hour of reaction showed a high decolorization rate compared with longer reactions times, especially for CV decolorization; as an example, the decolorization of CV with EL from grape seeds culture, attained 80% after 48 hours, but 40% decolorization was determined after the first hour of reaction.

**Figure 5:**
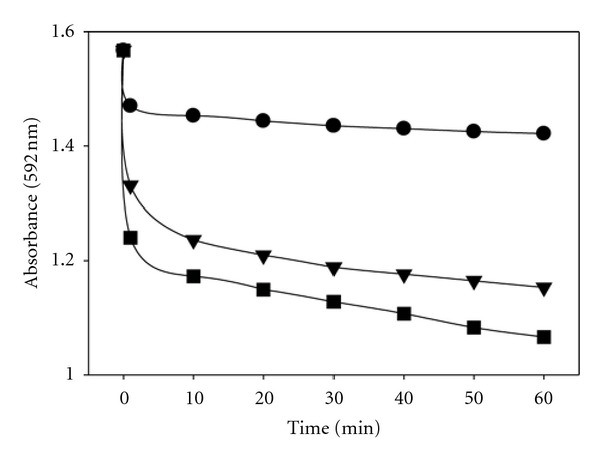
Decolorization of CV dye with UR (nonprotein fraction of extracellular liquid; MW < 10 KDa) obtained after culture of *T. versicolor* with several additives: *∙* wheat straw, ■ grape stalks, and *▼* grape seeds.

Therefore, UR from wheat straw, grape stalks, and grape seed was tested as decolorization agent of CV dye. The results of these experiments showed that URs of the several cultures were able to decolorize CV in different extent depending on the lignocellulosic residue employed in the culture (Figure 5). The common aspect is that the main positive decolorization effect is produced in the moment of UR addition to the reaction mixture, and then the decolorization is produced at a lower and constant rate for the rest of the treatment. Some results should be remarked: URs form grape stalks and grape seeds achieved a decolorization degree of 32% and 23%, respectively, after just one hour of reaction. This means that the oxidation potential of the nonprotein fraction is considerable and may play an important role in decolorization experiments depending on the dye used as substrate, since CV is sensible to the LMWM whereas PR is not clearly decolorized.

### 3.3. Effect of Organic Acids in Decolorization

Ligninolytic fungi usually secrete organic acids not only as a stabilization agent of enzymes but also as a possible method to produce radicals able to oxidize lignin in the very first moments of wood degradation. Some reports concerning the use of fungal laccases for the bioconversion of simple organic compounds into active compounds imply the suitability of these enzymes as regioselective reagents [26]. Actually, some roles of the organic acids have been described: Mn^3+^ stabilization after its production by manganese peroxidases [27], avoiding compound III (inactive enzyme) formation in lignin peroxidase redox cycle [28], hydroxyl radicals generation from oxalate [29] or from malonate oxidation by Mn^3+^ [30], electron source for Fe^3+^ reduction producing superoxide radicals [31]. Therefore, the presence of organic acids could explain the difference obtained by the several ELs tested in this work and the URs decolorization capability. The amount of organic acids produced by *T. versicolor* in the several cultures performed is showed in Table 2. Oxalic acid was the only organic acid that could be determined in a significant concentration. Culture performed with lignocellulosic residues showed a 10-fold concentration of this organic acid in comparison with the control culture carried out without them. Similar concentrations have been described by other authors [31, 32]. It seems that no relationship between oxalic acid concentration and decolorization potential of ELs could be detected, since different decolorization capability was obtained but there is no important difference in oxalic acid concentration. Nevertheless, the effect of organic acids in decolorization by laccase was studied in order to be able to undoubtedly discard their possible role in this degradation reaction.

**Figure 6:**
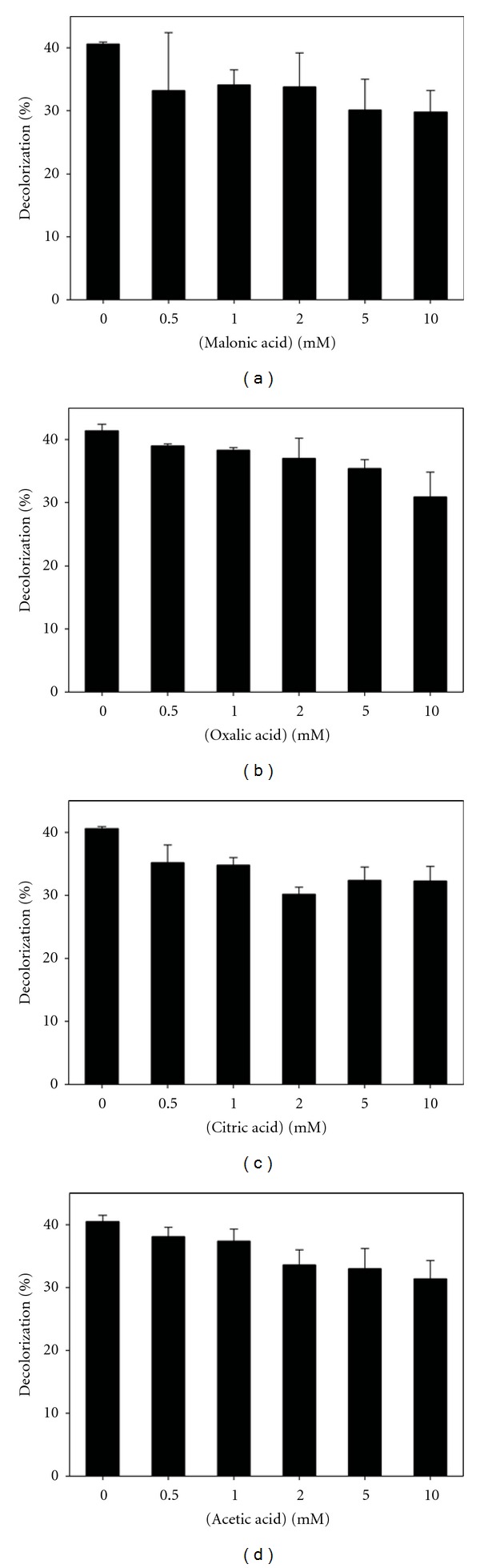
Decolorization of CV dye by laccase in presence of organic acids: malonic (a), oxalic (b), citric (c), and acetic (d) acids.

In Figure 6, the main results of this study are presented. As seen in Figure 6, the organic acids tested seemed to inhibit the decolorization of CV, even the decolorization inhibition was increased when organic acid concentration was augmented. Similar results were obtained for the four organic acids tested, suggesting that the presence of organic acids cannot explain neither the different decolorization capability of the several ELs produced, neither the nonenzymatic decolorization of CV carried out by URs. After carrying out similar experiments with PR dye, a possible positive effect was observed for high organic acid concentrations (data not shown). This result just could be interpreted as a possible mediator effect performed by the organic acids, but cannot be related with the nonenzymatic decolorization capability, since PR was not seriously decolorized by the URs. Moreover, this aforementioned possible mediator effect was observed at high acid organic concentrations (<10 mM) that were not detected in the ELs of *T. versicolor* cultures.

## 4. Conclusion

The decolorization of PR and CV was carried out with several enzymatic and nonenzymatic fractions from *T. versicolor* cultures. These two dyes were differentially decolorized suggesting the existence of enzymatic and nonenzymatic factors in the degradation reaction, suggesting a limited oxidative potential of the nonenzymatic factor. Among the compounds produced with molecular size lower than 10 KDa are outstanding the peroxides. The decolorization test with peroxides inhibition resulted in the disappearance of such nonenzymatic factor. Moreover, extracellular liquids from several cultures of *T. versicolor* in presence of lignocellulosic residues presented different decolorization ability as well. The employment of such residues offers several advantages from both economic and environmental points of view, but also as a method for producing extracellular liquids with improved dye decolorization capacity, although these differences are not due to the organic acids presented in the extracellular liquids.

## Figures and Tables

**Table 1:** Decolorization of crystal violet (CV) and phenol red (PR) with different enzymatic fractions and addition of catalase in reaction mixture.

Enzymatic fraction	Initial decolorization rate (ΔAbs/min)		Decolorization (%) after 6 days	
	CV	PR	CV	PR
Control	0.001	0.005	3	0
EL	0.242	0.101	93	67
EC	0.233	0.036	91	50
UR	0.017	0.000	42	0
EL + catalase	0.292	0.009	97	85
UR + catalase	0.000	0.000	12	0

**Table 2:** Oxalic acid concentration of cultures of *T. versicolor* performed with several lignocellulosic residues.

Culture	Control	Wheat straw	Corn cobs	Grape seeds	Grape stalks
Oxalic acid (mM)	0.14	1.15	1.25	1.34	1.35
